# Efeitos Agudos da Bebida Energética sobre Parâmetros Autonômicos e Cardiovasculares em Indivíduos com Diferentes Capacidades Cardiorrespiratórias: Um Ensaio Controlado, Randomizado, Crossover e Duplo Cego

**DOI:** 10.36660/abc.20210625

**Published:** 2022-07-28

**Authors:** Andrey Alves Porto, Luana Almeida Gonzaga, Cicero Jonas R Benjamim, Carlos Roberto Bueno, David M. Garner, Luiz C.M Vanderlei, Celso Ferreira, Vitor Engrácia Valenti

**Affiliations:** 1 Departamento de Fisioterapia Faculdade de Ciências e Tecnologias UNESP Presidente Prudente SP Brasil Departamento de Fisioterapia - Faculdade de Ciências e Tecnologias, UNESP, Presidente Prudente, SP - Brasil; 2 Centro de Estudos do Sistema Nervoso Autônomo UNESP Marília SP Brasil Centro de Estudos do Sistema Nervoso Autônomo (CESNA), UNESP, Marília, SP - Brasil; 3 Departamento de Medicina Interna Faculdade de Medicina de Ribeirão Preto Universidade de São Paulo Ribeirão Preto SP Brasil Departamento de Medicina Interna, Faculdade de Medicina de Ribeirão Preto, Universidade de São Paulo, Ribeirão Preto, SP - Brasil; 4 Escola de Educação Física de Ribeirão Preto Universidade de São Paulo Ribeirão Preto SP Brasil Escola de Educação Física de Ribeirão Preto, Universidade de São Paulo (EEFERP/USP), Ribeirão Preto, SP - Brasil; 5 Departamento de Ciências Biológicas e Médicas Faculdade de Saúde e Ciências da Vida Oxford Brookes University Oxford Reino Unido Grupo de Pesquisa Cardiorrespiratória, Departamento de Ciências Biológicas e Médicas, Faculdade de Saúde e Ciências da Vida, Oxford Brookes University, Headington Campus, Oxford - Reino Unido; 6 Departamento de Medicina UNIFESP São Paulo SP Brasil Departamento de Medicina, Universidade Federal de São Paulo, UNIFESP, São Paulo, SP - Brasil

**Keywords:** Bebidas Energéticas, Suplementos Nutricionais, Sistema Nervoso Autônomo, Sistema Cardiovascular

## Abstract

**Fundamento:**

Tem-se sugerido que o consumo de bebidas energéticas (BEs) possa afetar a atividade cardiovascular.

**Objetivos:**

Investigar os efeitos agudos da ingestão de BE sobre a variabilidade da frequência cardíaca (VFC) recuperação cardiovascular após exercício aeróbico moderado em homens de diferentes capacidades cardiorrespiratórias.

**Métodos:**

Este é um estudo randomizado, duplo cego, crossover, controlado por placebo. Vinte e oito jovens adultos foram divididos em dois grupos de acordo com o pico de consumo de oxigênio (pico de VO2): (1) pico de VO2 alto (AO) – pico de VO2 > 52,15 mL/Kg/min, e (2) pico de VO2 baixo (BO) - pico de VO2 <52,15 mL/Kg/min. Os indivíduos de ambos os grupos foram submetidos a dois protocolos de exercícios em ordem aleatória: exercício moderado aeróbico (60% de pico de VO2) após a ingestão de 250 mL de água (protocolo placebo) ou 250 mL de BE (protocolo BE). Durante os testes de exercício, foram registrados valores de parâmetros cardiorrespiratórios e de VFC.

**Resultados:**

Foram observadas diferenças significativas para o índice de LF (unidades normalizadas) entre “repouso” e “Rec1” nos grupos de AO e BO durante o protocolo BE. Para a razão LF/HF, foram observadas diferenças significativas entre “repouso” e Rec1 nos grupos AO e BO nos protocolos BE.

**Conclusão:**

A ingestão aguda de BE retardou a recuperação da frequência cardíaca após o exercício em indivíduos com capacidade cardiorrespiratória baixa e indivíduos com capacidade cardiorrespiratória alta.

## Introdução

As bebidas energéticas (BEs) são amplamente consumidas no meio esportivo para melhorar o estado de alerta e o desempenho, e seu uso é principalmente atribuído ao seu teor de cafeína.^1 , 2^ De acordo com o Comitê Olímpico Internacional^3^ e a Sociedade Internacional de Nutrição Esportiva,^4^ a cafeína é considerada um suplemento ergogênico capaz de aumentar o desempenho físico durante o exercício.^3 , 4^ Presume-se que outros componentes das BE (por exemplo, vitaminas e minerais) tenham sinergismo com a cafeína e a taurina, podendo, assim, potencializar seus efeitos. No entanto, essas questões não foram totalmente elucidadas.^5^

Muitos estudos foram realizados sobre os potenciais efeitos das BEs no sistema cardiovascular.^6^ Até o momento, os resultados mostram que um consumo modesto de BEs, o que corresponde a 200 mg de cafeína, não causa risco à saúde cardiovascular. No entanto, o consumo agudo de aproximadamente 1000mL de BE foi associado a um aumento nos efeitos cardiovasculares adversos (por exemplo, intervalo QT prolongado e taquicardias).^6 , 7^

Ainda, a literatura científica tem destacado que o uso de estimulantes pode aumentar o risco de eventos cardíacos adversos durante e após o exercício.^7^ A redução da frequência cardíaca (FC) após o exercício tem sido demonstrado como um preditor importante de eventos cardíacos adversos e mortalidade.^8^ Sua análise tem sido cada vez mais utilizada como uma técnica não invasiva, mas confiável, para avaliar a adaptação do sistema nervoso autonômico (SNA) (reativação vagal) a várias condições.^9^

A variabilidade da FC (VFC) avalia as flutuações nos intervalos entre batimentos cardíacos consecutivos (intervalos RR), o que reflete a função do SNA.^9^ Indivíduos sadios e fisicamente ativos apresentam uma rápida recuperação da FC após o exercício, o que permite adaptação adequada do SNA e baixo risco cardiovascular.^10^ Assim, o uso de compostos pode atrasar a recuperação autonômica pós-exercício, rompendo, assim, o controle autonômico da FC.^10^

Evidência científica tem mostrado que o consumo moderado de cafeína isolada (por exemplo, 3-6mg/kg ou 300-400mg em uma única dose) pode atrasar a recuperação da FC após o exercício.^11 , 12^ Recentemente, foi publicado que a cafeína exerce maiores efeitos sobre indivíduos com uma baixa capacidade cardiorrespiratória, medida pelo VO2max, no que diz respeito à recuperação da FC após o exercício.^13^

Até o presente momento, estudos que avaliaram os efeitos das BEs sobre a recuperação da FC não os comparou entre populações de diferentes perfis cardiorrespiratórios.^14 - 17^ Uma dose modesta de aproximadamente 250mL de BE parece não afetar a recuperação da FC após o exercício em indivíduos treinados.^14 - 16^ Porém, nenhum estudo levou em consideração a capacidade cardiorrespiratório dos indivíduos e, portanto, ainda existe uma lacuna na literatura.

Assim, este estudo teve como objetivo avaliar os efeitos agudos da ingestão de BE sobre a recuperação da FC e cardiovascular após exercícios aeróbicos moderados em indivíduos do sexo masculino com diferentes capacidades cardiorrespiratórias. Os participantes foram divididos de acordo com o pico de consumo de oxigênio (pico de VO2 pico).^18^

## Métodos

Este estudo foi conduzido de acordo com os padrões definidos pelo grupo CONSORT ( *Consolidated Standards of Reporting Trials* ). Este é um estudo crossover, duplo cego, randomizado, e controlado com placebo. O estudo foi avaliado e aprovado pelo comitê de ética da UNIFESP (número do registro: CEP-2200/11). Todos os pacientes que concordaram em participar do estudo assinaram um termo de consentimento. Os detalhes dos protocolos experimentais estão registrados no Clinical Trials.gov (primeira publicação em 28 de setembro de 2016) (número de protocolo NCT02917889, https://clinicaltrials.gov/ct2/show/NCT02917889).

### Participantes

O estudo foi realizado com jovens adultos fisicamente ativos recrutados por mídia social. Excluímos indivíduos que não eram considerados fisicamente ativos segundo o questionário internacional de atividade física (IPAQ, *International Physical Activity Questionnaire* ).

### Avaliação inicial

Os indivíduos foram primeiramente entrevistados para obtenção de dados como idade (anos), peso corporal (Kg), altura (cm), e índice de massa corporal (Kg/m^2^ ). As medidas antropométricas foram realizadas de acordo com recomendações previamente publicadas.^19^

### Intervenções

O protocolo experimental consistiu em três fases, com um intervalo mínimo de 48 horas para permitir adequada recuperação dos indivíduos.

O estudo foi conduzido entre 17h30 e 21h30 para padronização das flutuações circadianas, em uma sala silenciosa com umidade entre 60 e 70%, e temperatura entre 23^o^C e 24^o^C.^20^ Os indivíduos foram orientados a se absterem de bebida alcoólica ou de realizarem exercícios exaustivos 24 horas antes de cada seção e evitar o consumo de alimentos e bebidas cafeinadas 24 horas antes do procedimento experimental. Os indivíduos foram aconselhados a vestirem roupas confortáveis apropriadas para se exercitarem, e a consumirem uma refeição leve duas horas antes dos procedimentos.

Seguindo recomendações do Colégio Americano da Medicina do Esporte (ACSM),^21^ para evitar desidratação dos participantes durante o exercício,^22^ os participantes foram orientados a beberem 500mL de água duas horas antes dos testes.

Na primeira fase do estudo, o VO2max de cada participante foi definido. Na segunda fase, os indivíduos seguiram o protocolo placebo (PP) (250mL de água) ou o protocolo de BE (BP) (250mL de BE) 15 minutos antes do exercício. Na terceira fase, os participantes seguiram o protocolo contrário ao seguido na fase anterior. Um pesquisador independente que não participou da coleta dos dados forneceu as bebidas. Tanto os pesquisadores como os participantes eram cegos para a sequência das intervenções.

A BE (250mL) tinha um valor energético de 45 kcal e era composta de 11,2 g de carboidratos, 80 mg de sódio, 32 mg de cafeína, 400 mg de taurina, 4,6 mg de niacina, 2 mg de ácido pantotênico, 0,5 mg de vitamina B6, 0,4 mg de vitamina B12, 240 mg de glucoronolactona, e 20 mg de inositol.^16^

A intensidade dos exercícios aeróbicos em todos os estágios foi prescrita com base no VO2max de cada participante. O teste da esteira teve uma duração total de 30 minutos. Primeiro, os indivíduos caminharam sobre uma esteira a uma velocidade de 5Km/h por cinco minutos para aquecimento; em seguida, a velocidade foi aumentada ao correspondente a 60% do VO2max por 25 minutos. Por fim, os indivíduos descansaram por 60 minutos (recuperação) na posição supina.

#### Variáveis cardiorrespiratórias

O teste para determinar o VO2max foi realizado em uma esteira (TPEE; Inbrasport ATL 2000) usando o protocolo de Bruce.^23^ Os indivíduos permaneceram em repouso sobre a esteira, em posição ortostática, para estabilização dos valores basais. Em seguida, o teste de estresse foi iniciado, com aumento progressivo na carga aumentando-se a inclinação e a velocidade da esteira a cada três minutos. Reforço verbal foi dado na tentativa de se obter máximo esforço físico. O teste foi interrompido por exaustão ou qualquer anormalidade clínica ou eletrocardiográfica.

Durante o teste, a FC e a percepção subjetiva de esforço foram monitoradas ao final de cada estágio pela escala de Borg para dor e esforço percebidos.^24^ Para que o teste fosse reconhecido como máximo, os indivíduos deveriam atingir 90% da FC máxima, calculada previamente (220 – idade).^25^

A análise dos gases expirados foi realizada utilizando o sistema comercial Quark PFT (Comend, Roma, Itália), e o pico de VO2 foi definido como o VO2max mais alto alcançado durante o teste.

Os indivíduos foram separados em dois grupos com base no pico de VO2 mediano:

1. Grupo com pico de VO2 alto (AO), composto de indivíduos com pico de VO2 > 52,15 mL/kg/min, e
2. Grupo com pico de VO2 baixo (BO), composto de indivíduos com pico de VO2 < 52,15 mL/kg/min.

#### Parâmetros cardiovasculares

Os parâmetros cardiovasculares foram medidos com os indivíduos na posição supina. Pressão arterial sistólica (PAS) e pressão arterial diastólica (PAD) foram obtidas por ausculta com estetoscópio (Littman Classic II^®^, St. Paul, EUA) e esfigmomanômetro aneroide (Welch Allyn Tycos^®^, New York, EUA) no braço esquerdo. A FC foi medida usando um monitor Polar RS800CX^®^. A taxa respiratória (TR) foi determinada contando-se a respiração de cada participante por um minuto, sem seu conhecimento, de modo que não alterasse seu padrão respiratório. A saturação de oxigênio (SpO_2_) foi medida por oximetria de pulso (PM-50 Mindray^®^).

#### Análise da VFC

A VFC foi medida seguindo-se as recomendações da Força Tarefa da Sociedade Europeia de Cardiologia ( *Task Force of the European Society of Cardiology* ) e da Sociedade Norte-Americana de Estimulação Cardíaca e Eletrofisiologia ( *North American Society of Pacing and* Electrophysiology).^26^ O transmissor de FC foi usado no peito e o receptor Polar RS800CX colocado no punho esquerdo. O padrão de VFC foi registrado a cada batimento. Foram selecionados os 256 intervalos RR estáveis consecutivos de cada registro. Os dados passaram por filtragem digital e manual para eliminar artefatos e batimentos ectópicos prematuros. Somente as séries com um excesso de 95% de batimentos sinusais foram incluídos na análise.

O índice tempo-domínio da VFC foi determinado pela raiz quadrada média das diferenças sucessivas (RMSSD) e o desvio padrão dos intervalos RR normais (SDNN). O índice domínio-frequência foi avaliado pelo componente alta frequência (HF) da densidade espectral (0,15-0,4 Hz), pelo componente baixa frequência (LF) em milissegundos ao quadrado e unidades absolutas, e razão LF/HF (ms^2^). O gráfico de Poincaré foi construído usando os índices: desvio padrão da variabilidade instantânea a cada batimento (SD1) e o desvio padrão da variabilidade contínua a cada batimento, em longo prazo (SD2). Esses índices foram computados usando o programa de análise Kubios HRV^®^.

#### Medida dos parâmetros

A FC, a TR, a PAS, a PAD e a SpO2 foram registradas nos seguintes tempos: repouso – 15 minutos após a ingestão de BE ou controle – e durante a recuperação – minutos 1, 3, 5, 7, 10, 20, 30, 40, 50 e 60 após exercício.

Os índices da VFC foram medidos nos seguintes tempos: “repouso” (15-20 minutos de repouso após ingestão de BE ou placebo; e durante a “recuperação”: Rec1 (zero a cinco minutos), Rec 2 (cinco a 10 minutos), Rec3 (15 a 20 minutos), Rec4 (25 a 30 minutos), Rec5 (35 a 40 minutos), Rec6 (45 a 50 minutos), e Rec7 (55 a 60 minutos).

#### Tamanho amostral

O tamanho amostral foi calculado com base em um estudo prévio,^22^ que nos deu a magnitude da diferença, e nós calculamos o índice RMSSD como referência. Determinados um desvio padrão de 16,2 ms e a magnitude da diferença foi 11ms. O tamanho amostral calculado foi de um mínimo de 14 indivíduos por grupo, com um risco alfa de 5% e risco beta de 80%.

## Análise estatística

A análise e o registro dos dados foram conduzidos seguindo-se as recomendações de Laborde et al.^27^ A normalidade dos dados foi testada pelo teste de Shapiro-Wilk. Para comparar as variáveis cardiovasculares e VFC, realizamos a análise de variância (ANOVA) de medidas repetidas, seguida do teste de Bonferroni para distribuições paramétricas ou o teste de Friedman seguido do teste de Dunn para distribuições não paramétricas. Valores de p <0,05 foram considerados significativos. As análises foram realizadas usando o programa IBM SPSS Statistics, versão 22.0 (SPSS Inc., Chicago, IL, EUA).

### Randomização e avaliação do desfecho

Visando minimizar o viés de seleção, os participantes e os pesquisadores não eram informados quanto à ordem dos procedimentos. Um pesquisador que não participou do estudo conduziu a alocação aleatória dos participantes às intervenções. Pesquisadores especializados na área, que não participaram da coleta de dados, foram convidados a avaliar o desfecho. Assim, os avaliadores do desfecho eram cegos, diminuindo a susceptibilidade do estudo ao viés de detecção. Ainda, todos os desfechos foram relatados na íntegra, diminuindo a chance de viés de publicação (ou de relato).

## Resultados

**Figura 1:**
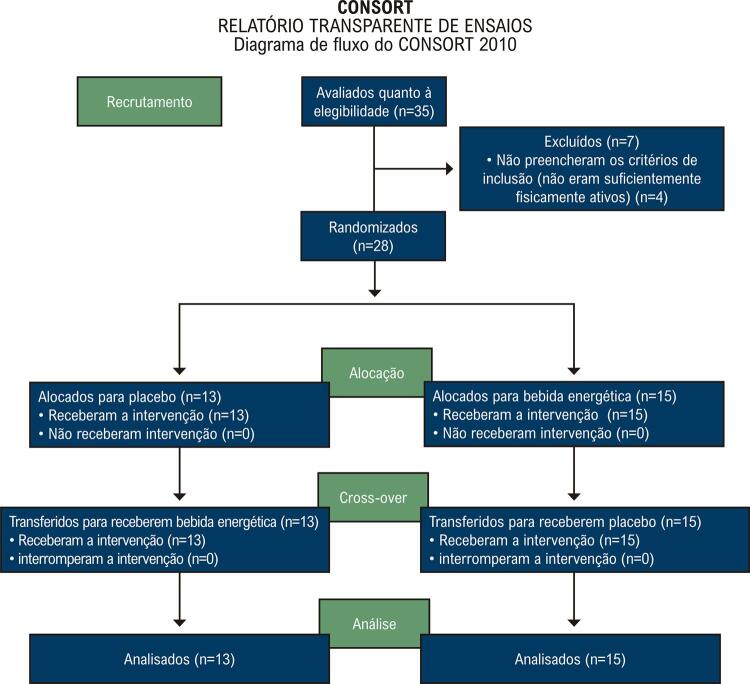
Diagrama de fluxo do CONSORT.

Um total de 35 homens foram avaliados quanto à elegibilidade; 28 preencheram os critérios de inclusão e completaram o estudo ( Figura 1 ).

A Tabela 1 descreve as características antropométricas e as respostas obtidas no teste de esforço máximo para os grupos com o pico de VO2 (AO) mais alto e o pico de VO2 mais baixo (BO).

**Tabela 1:** Características antropométricas e valores de pico de VO2 dos participantes do estudo

	Pico de VO2 alto		Pico de VO2 baixo		Valor de p

	Média ± DP	Min - Máx	Média ± DP	Min - Máx	
Idade (anos)	22,93 ± 2,62	[18 - 26]	25,29 ± 3,07	[21 - 29]	0,038*
Altura (m)	1,78 ± 0,08	[1,68 - 1,94]	1,81 ± 12,52	[1,65 – 1,93]	0,286
Peso corporal (kg)	77,55 ± 6,92	[60 - 96]	89,48 ± 12,52	[63,30 – 107,50]	0,014*
IMC (Kg/m^2^)	24,46 ± 2,56	[20,05 - 29,41]	27,12 ± 3,07	[19,94 - 27,70]	0,012*
Pico VO2 (ml/kg/min)	60,14 ± 6,43	[52,40 - 77,77]	41,76 ± 10,14	[23,03 – 29,94]	<0,001*

*m: metros; kg: quilograma; IMC: índice de massa corporal; Min: mínimo; Máx: máximo.*

**Figura 2:**
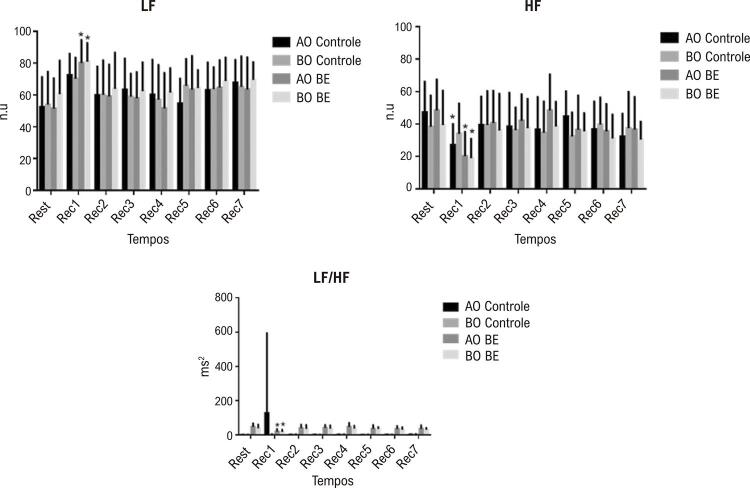
Resposta dos índices da variabilidade da frequência cardíaca no domínio da frequência em repouso e durante a recuperação do exercício nos grupos de indivíduos com alto pico de VO2 (AO) e baixo pico de VO2 (BO) recebendo bebida energética (BE) ou placebo (controle). LF: baixa frequência; HF: alta frequência.

Em relação ao domínio da frequência e os índices da VFC, detectamos um efeito do tempo (p=0,0001). Não foi detectado efeito de interação do protocolo para LF (n.u.) (p=0,880), HF (n.u.) (p=0,163) e LF/HF ms^2^ (p=0,086). Ainda, não observamos efeito do protocolo sobre LF (n.u.) (p=1,000), HF (n.u.) (p=0,675) e LF/HF (p=0,531). Para o índice LF (n.u,), diferenças significativas foram observadas entre repouso e Rec1 nos grupos AO e BO nos protocolos de BE. Houve diferenças significativas em AF (unidades normalizadas, u.n.) entre repouso e Rec1 para AO no PP, e para AO e BO durante o PB. Em relação à razão LF/HF, diferenças significativas foram encontradas entr repouso e Rec1 nos grupos AO e BO durante o PB. As respostas dos índices do domínio da frequência da VFC estão apresentadas na Figura 2 .

SDNN e SD2 mostraram diferenças significativas nos efeitos do tempo (SDNN: p=0,0001; SD2: p=0,0001) interação do protocolo (SDNN: p<0,0001; SD2: p=0,0002) e somente para SDNN foi diferente entre protocolos (SDNN: p=0,015; SD2 p=0,061). Foram observadas diferenças significativas somente para o índice SDNN (SDNN: p=0,015; SD2 p=0,061). Diferenças significativas foram observadas no índice SDNN entre o repouso e Rec1 para o grupo BO durante o protocolo placebo, e no índice RMSSD entre repouso e Rec1 nos protocolos placebo e BE.

**Figura 3:**
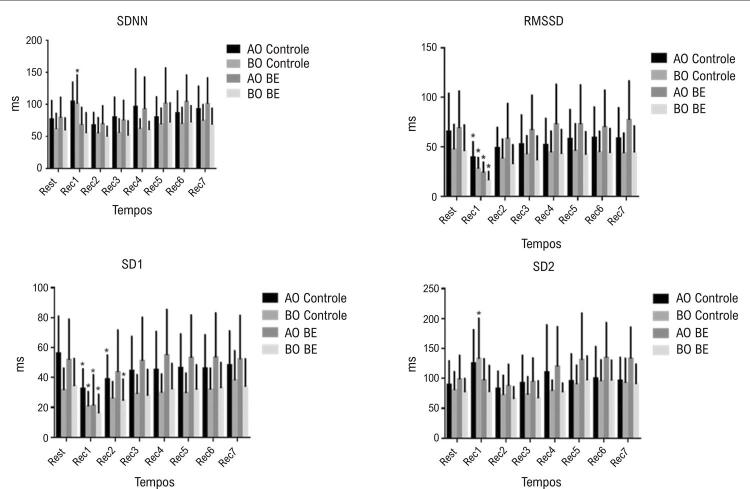
Resposta dos índices da variabilidade da frequência cardíaca no domínio do tempo em repouso e durante a recuperação do exercício nos grupos de indivíduos com alto pico de VO2 (AO) e baixo pico de VO2 (BO) recebendo bebida energética (BE) ou placebo (controle).

Em relação ao RMSSD e SD1, observamos diferenças significativas nos efeitos do tempo (RMSSD: p<0,0001; SD1: p<0,0001), interação do protocolo (RMSSD: p=0,009; SD1: p=0,036), e entre protocolos (RMSSD: p=0,025; SD1=0,010). Mudanças significativas para o domínio do tempo foram observadas entre repouso e Rec1 para o índice RMSSD e índice SD1 para todos os protocolos. Diferenças significativas para o domínio tempo foram observadas entre repouso e Rec2 para AO no protocolo placebo e BO no protocolo BE para SD1. A Figura 3 mostra a resposta da VFC no domínio do tempo em repouso e durante a recuperação do exercício.

**Figura 4:**
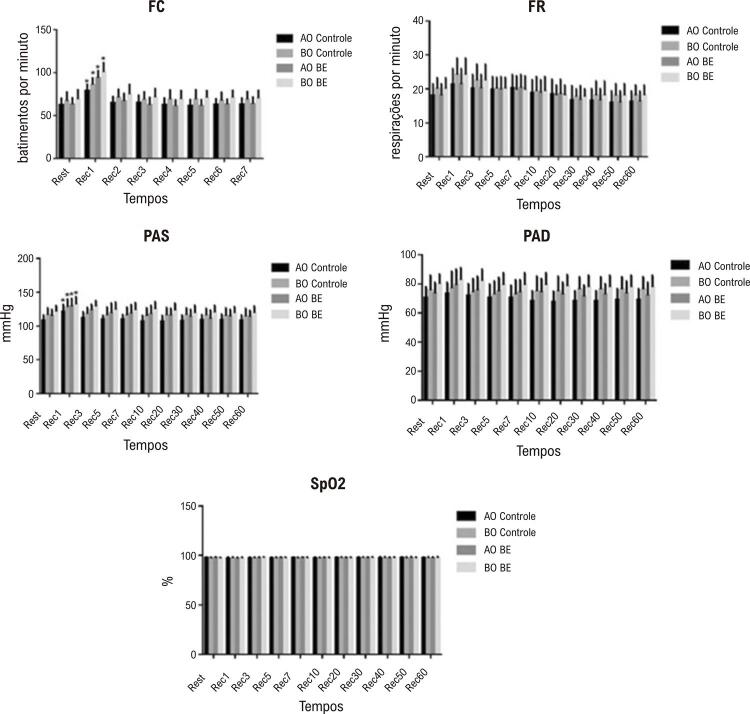
Parâmetros cardiorrespiratórios em repouso e durante recuperação do exercício nos grupos de indivíduos com alto pico de VO2 (AO) e baixo pico de VO2 (BO) recebendo bebida energética (BE) ou placebo (controle). FC: frequência cardíaca; FR: frequência respiratória; PAS: pressão arterial sistólica; PAD: pressão arterial diastólica.

Quanto aos parâmetros cardiorrespiratórios, observamos um efeito do tempo (p=0,0001) para FC, TR, PAS, PAD (p=0,0001), e nenhum efeito foi observado na SpO2 (p=0,188). Nenhum efeito de interação de protocolo significativo foi observado para PAS, PAD, TR ou SpO2 (PAS: p=0,424; PAD: p=0,259; TR: p=0,340; SpO2: p=0,346), mas um efeito significativo foi observado para FC (p<0,0001). Diferenças significativas na FC e PAD foram observadas no domínio do tempo entre repouso e Rec1 para todos os protocolos. A Figura 4 apresenta resposta dos parâmetros cardiorrespiratórios em repouso e durante a recuperação do exercício.

## Discussão

Nosso estudo foi realizado para avaliar o impacto da ingestão de BE sobre a VFC e recuperação cardiovascular após o exercício em indivíduos com diferentes capacidades cardiovasculares. Como resultados principais, encontramos que a BE antes do exercício não teve efeito sobre PAS, PAD, SpO2 ou TR, e retardou a recuperação da LF e da LF/HF após o esforço.

Constituintes tais como cafeína, taurina, glucoronolactona, vitaminas B, guaraná, ginseng, ginkgo biloba, l-carnitina, açúcares, antioxidantes e elementos traços são geralmente encontrados nas BEs.^28^ A cafeína estimula o sistema nervoso central via ativação do sistema simpático (medula adrenal), elevando a pressão sanguínea em situações de estresse psicológico^29^ e fisiológico, como por exemplo, exercício físico.^30 , 31^

Ajustes cardiovasculares são necessários para a manutenção de perfusão adequada a outros órgãos.^32^ Quando o exercício é iniciado, o comando central ajusta o barorreflexo arterial, resultando em uma condução parassimpática diminuída, e leve redução na atividade do SNA devido a um retorno venoso nessa fase.^33^

A elevação da amplitude reflexiva por meio do aumento precoce na FC é causada por um aumento na carga sobre os barorreceptores pulmonares, o que permite que o sistema nervoso parassimpático interrompa sua atividade cardíaca. À medida que a carga de exercícios aumenta, o comando central aumenta e readapta o barorreflexo arterial. Assim, ocorre uma depressão da resposta reflexa do sistema parassimpático, aumento no sistema nervoso simpático, aumentando a FC e força de contração cardíaca.^34^

Há registros na literatura científica que indicam uma íntima conexão entre BE e alterações no sistema cardiovascular. BEs suprimem o sistema nervoso parassimpático e/ou aumentam o sistema nervoso simpático em jovens obesos,^35^ aumentam a PAS,^36^ altera a VFC não linear em adultos jovens,^37^ e retardam a FC e a VFC pós-exercício quando misturadas com bebidas alcoólicas.

Recentemente, nosso grupo relatou que a BE não é capaz de adiar a recuperação da FC após o exercício.^15^ No estudo citado, 29 homens sadios com idade entre 18 e 30 anos realizaram exercício aeróbico após ingerirem BE ou placebo. Houve uma importante redução na VFC nos primeiros cinco minutos pós-exercício em ambos os protocolos. Portanto, a principal conclusão foi que a BE não foi capaz de influenciar a recuperação da FC após o exercício.^15^ Em outro estudo com protocolos semelhantes, An et al.^16^ não detectaram diferenças nesses parâmetros entre as duas intervenções, sugerindo nenhum efeito significativo da BE.

Em outro ensaio clínico, crossover, randomizado, controlado com placebo, 15 adultos jovens (oito homens), fisicamente ativos, foram avaliados quanto aos efeitos da ingestão de BE.^17^ Após jejum de oito horas, os participantes consumiram BE padrão (2mg/kg de cafeína) ou placebo com sabor similar. Após exercício aeróbico submáximo por 30 minutos, os indivíduos foram induzidos à fadiga pedalando por 10 minutos a 80% do limiar ventilatório. A FC de repouso foi maior quando os indivíduos ingeriam a BE, em comparação à ingestão de placebo (BE: 65+10bpm vs. Placebo: 58+8bpm, p=0,02), embora os índices da VFC (RMSSD, SDNN, PNN50, FC, LF e LF/HF) mantiveram-se sem alteração significativa.^17^

No estudo duplo-cego, crossover, contrabalanceado e controlado com placebo de Clark et al.^14^ 17 (10 mulheres) adultos jovens foram expostos a um teste de exercício gradativo de exaustão, em uma bicicleta, após a ingestão de 140mg de cafeína ou placebo. Os parâmetros da VFC foram registrados antes, durante e após 15 minutos de exercício. Foram observados aumentos importantes na FC e no RMSSD no grupo BE durante o exercício. Uma análise entre os sexos revelou alterações nos valores iniciais de RMSSD e no grau de diminuição. O consumo de BE foi capaz de afetar as respostas cardíacas autonômicas durante exercício de intensidade leve, moderada e alta, e tais mudanças eram diferentes entre homens e mulheres. No entanto, nenhuma alteração na recuperação da FC foi observada após o exercício com a ingestão de BE.

É crucial enfatizar que esses estudos não levaram em consideração a capacidade cardiorrespiratória dos indivíduos. Um estudo mais recente^13^ avaliou o impacto da cafeína sobre a recuperação da FC pós-exercício em homens com diferentes VO2. Os autores separaram os participantes (adultos jovens) em dois grupos, de acordo com seus valores de VO2: (1) VO2 alto (AO): 16 voluntários, pico de VO2> 42,46 mL/Kg/min; e (2) VO2 baixo (BO): 16 voluntários, VO2 <42,46 mL/Kg/min). Os indivíduos participaram de dois protocolos, que incluíram a ingestão de cápsulas contendo 300 mg de amido (protocolo placebo) ou 300 mg de cafeína (protocolo cafeína). Após a ingestão da cápsula, os participantes descansaram por 15 minutos, e em seguida foram submetidos a 30 minutos de exercício na esteira a 60% do pico de VO2. Os índices de VFC nos domínios do tempo e da frequência revelaram alterações significativas de RMSSD e SDNN na recuperação entre os grupos (p<0,001). Ajustes marcantes foram observados (repouso *versus* recuperação) nos primeiros cinco minutos de recuperação pós-exercício para o grupo BO no protocolo placebo, e entre cinco e 10 minutos de recuperação no grupo BO no protocolo cafeína. Em nosso estudo, desvios importantes foram detectados somente nos primeiros cinco minutos dos indivíduos AO em ambos os protocolos. Esses dados corroboram que a cafeína atrasa a recuperação parassimpática após o exercício em indivíduos com menor capacidade cardiorrespiratória.^13^

Em relação aos parâmetros cardiorrespiratórios, não foram detectadas alterações significativas que pudessem sugerir diferentes efeitos da BE em indivíduos com capacidades cardiorrespiratórias diferentes. Esse resultado corrobora o estudo de An et al.,^16^ em que não foram observadas alterações significativas na FC e na pressão sanguínea durante a recuperação após exercício máximo, após a ingestão de BE em diferentes concentrações (1,25-2,5 mg/Kg).

O efeito da BE sobre o sistema cardiovascular parece estar relacionado à dose ingerida. No estudo de Shah et al.,^35^ o consumo de BE em altas doses (946 mL) resultou em um aumento significativo e prolongado no intervalo QTc, na PAS e PAD em comparação a placebo em indivíduos jovens sadios.

Em relação aos parâmetros que refletem o componente respiratório, como a SpO2 e a FC, não observamos diferenças significativas em nosso estudo. Em ambos os protocolos, todos os indivíduos apresentaram valores adequados dessas variáveis, o que seria esperado para indivíduos sadios sem diagnóstico de doença cardiopulmonar.^11^

Por fim, considerando que detectamos um pequeno atraso na recuperação da FC em ambos os grupos que ingeriram a BE, nossos dados chamam a atenção para os indivíduos com doenças cardiovasculares e metabólicas que fazem uso de BEs (como um suplemento) antes da prática de exercício físico.

### Pontos fortes e limitações do estudo

Um dos pontos fortes deste estudo refere-se à sua metodologia. Embora não tenhamos avaliado as concentrações plasmáticas de catecolamina ou atividade nervosa simpática, nós avaliamos a VFC, um método simples, confiável, não invasivo, e um marcador quantitativo importante para estimar a modulação autonômica da FC.^9^ A amostra era composta por jovens saudáveis, a fim de se evitar a influência de hormônios sexuais. Por isso, nossos resultados não podem ser estendidos a mulheres ou indivíduos em uso de medicamentos que possam afetar o SNA. No entanto, o delineamento do estudo e a realização de procedimentos rigorosos para evitar vieses de seleção, detecção, atrito, e relato reforçam nossos resultados. Nosso estudo fornece informação importante sobre os mecanismos relacionados ao impacto da BE sobre a recuperação pós-exercício.

## Conclusões

A ingestão aguda de BE atrasou a recuperação da FC após o exercício em indivíduos com baixa ou alta capacidade cardiorrespiratória.
