# Supplementary material for: Efeitos Agudos da Bebida Energética sobre Parâmetros Autonômicos e Cardiovasculares em Indivíduos com Diferentes Capacidades Cardiorrespiratórias: Um Ensaio Controlado, Randomizado, Crossover e Duplo Cego
Source: Arq Bras Cardiol. 2022 Jul 28;119(4):553–61. [Article in Portuguese] doi: 10.36660/abc.20210625 (PMC9563894; doi:10.36660/abc.20210625)
Supplement: Translation

# Translation: Acute Effects of Energy Drink on Autonomic and Cardiovascular Parameters Recovery in Individuals with Different Cardiorespiratory Fitness: A Randomized, Crossover, Double-Blind and Placebo-Controlled Trial

**Authors:** Andrey Alves Porto, Luana Almeida Gonzaga, Cicero Jonas R Benjamim, Carlos Roberto Bueno, David M. Garner, Luiz C.M Vanderlei, Celso Ferreira, Vitor Engrácia Valenti

**Affiliations:** 1 Brasil Departamento de Fisioterapia - Faculdade de Ciências e Tecnologias, UNESP, Presidente Prudente, SP - Brasil; 2 Brasil Centro de Estudos do Sistema Nervoso Autônomo (CESNA), UNESP, Marília, SP - Brasil; 3 Brasil Departamento de Medicina Interna, Faculdade de Medicina de Ribeirão Preto, Universidade de São Paulo, Ribeirão Preto, SP - Brasil; 4 Brasil Escola de Educação Física de Ribeirão Preto, Universidade de São Paulo (EEFERP/USP), Ribeirão Preto, SP - Brasil; 5 Reino Unido Grupo de Pesquisa Cardiorrespiratória, Departamento de Ciências Biológicas e Médicas, Faculdade de Saúde e Ciências da Vida, Oxford Brookes University, Headington Campus, Oxford - Reino Unido; 6 Brasil Departamento de Medicina, Universidade Federal de São Paulo, UNIFESP, São Paulo, SP - Brasil

**Keywords:** Energy Drinks, Dietary Supplements, Exercise, Autonomic Nervous System, Cardiovascular system

## Abstract

**Background:**

It has been suggested that the consumption of energy drinks (ED) may affect cardiovascular activity.

**Objectives:**

to investigate the acute effects of ED intake on heart rate variability (HRV) and cardiovascular recovery after moderate aerobic exercise in males with different cardiorespiratory capacities.

**Methods:**

This is a randomized, double-blind, crossover, placebo-controlled study. Twenty-eight young adults were split into two groups according to their peak oxygen consumption (VO2peak) values: (1) High VO2 peak (HO) - VO2 peak > 52.15 mL/kg/min, and (2) low VO2 peak (LO) - peak VO2 <52.15 mL/kg/min. Subjects of both groups underwent two exercise protocols in randomized order: moderate aerobic exercise (60% of VO2peak) following the intake of 250 mL of water (placebo protocol) or 250 mL of ED (ED protocol). During the exercise tests, values of cardiorespiratory and HRV parameters were recorded.

**Results:**

Significant differences were observed for the LF (normalized units) index between rest and Rec1 in HO energy and LO groups during the ED protocol. For the LF/HF ratio, significant differences were seen between rest and Rec1 in HO and LO during ED protocols.

**Conclusion:**

Acute ED intake delayed heart rate recovery after exercise in subjects with low and high cardiorespiratory fitness.

## Introduction

Energy drinks (EDs) are widely consumed in the sport environment to improve alertness and performance, and their use is mainly attributed to their caffeine content.^1 , 2^ According to the International Olympic Committee^3^ and the International Society of Sports Nutrition,^4^ caffeine is considered an ergogenic supplement capable of increasing physical performance during exercise.^3 , 4^ There is conjecture that other ED components (e.g., vitamins and minerals) have synergism with caffeine and taurine, and thereby may potentiate their effects. However, these issues have not been fully elucidated.^5^

Numerous studies have been performed to better understand the potential effects of EDs on the cardiovascular system.^6^ So far, it has been found that a modest consumption of EDs, corresponding to 200mg of caffeine, poses no risk to the cardiovascular health. Nevertheless, the acute consumption of approximately 1,000mL of ED was associated with an increase in adverse cardiovascular effects (e.g., prolonged QT interval and tachycardias).^6 , 7^

Still, the scientific research literature has highlighted that stimulants may increase the risk of adverse cardiac events during and after exercise.^7^ Heart rate (HR) slowing after exercise has been demonstrated to be an important predictor of adverse cardiac events and mortality.^8^ Its analysis has been increasingly used as a non-invasive, yet reliable technique to study the adaptation of the autonomic nervous system (ANS) (vagal reactivation) to various conditions.^9^

HR variability (HRV) evaluates the fluctuations in the intervals between consecutive heartbeats (RR intervals), which reflects the ANS function.^9^ Physically active and healthy subjects show rapid HR recovery from exercise, which allows adequate ANS adaptation and low cardiovascular risk.^10^ Thus, the use of compounds that delay the post-exercise autonomic recovery can lead to cardiac activity overload, thereby disrupting the autonomic control of HR.^10^

Scientific evidence has shown that the moderate consumption of caffeine alone (e.g., 3-6mg/kg or 300-400mg in a single dose) is permissible to delay HR recovery following exercise.^11 , 12^ Recently, it has been documented that caffeine has greater effects on individuals with a low cardiorespiratory capacity, measured by the maximum oxygen consumption (VO2max), concerning post-exercise HR recovery.^13^

So far, studies that evaluated the effects of EDs on HR recovery have not compared them between populations with different cardiorespiratory profiles.^14 - 17^ A modest dose of approximately 250mL of ED seems to have no effect on HR recovery after exercise in trained individuals.^14 - 16^ Even so, no study has considered the individuals’ cardiorespiratory capacity and, hence, there is still a gap in the literature.

Therefore, this study aimed to evaluate the acute effects of ED intake on HR and cardiovascular recovery after moderate aerobic exercise in young male adults with different cardiorespiratory capacities. Participants were divided according to their peak oxygen consumption (VO2 peak).^18^

## Methods

This study was reported according to the Consolidated Standards of Reporting Trials (CONSORT) statement. This is a crossover, randomized, double-blind and placebo-controlled trial. The study was evaluated and approved by UNIFESP ethics committee (registration number: CEP-2200/11). All participants signed an informed consent agreeing to participate in the study. The details of the experimental protocols are registered in the Clinical Trials.gov (first publication – September 28, 2016) (Protocol NCT02917889, https://clinicaltrials.gov/ct2/show/NCT02917889).

### Participants

The study was performed with healthy and physically active young adult males recruited via social media. We excluded subjects who were not considered physically active according to the International Physical Activity Questionnaire (IPAQ).^18^

### Initial assessment

The individuals were first interviewed to obtain data such as: age (years), body weight (Kg), height (cm), and body mass index (Kg/m^2^). Anthropometric measures were taken according to previously published recommendations.^19^

### Interventions

The experimental protocol consisted of three phases with an interval of at least 48 hours to allow adequate recovery of the subjects.

The study was performed between 17:30 and 21:30 to standardize circadian variations, in a quiet room with humidity between 60% and 70% and temperature between 23°C and 24°C.^20^ The subjects were told to refrain from drinking alcohol or performing exhaustive exercise 24 hours prior to each section and to avoid ingesting caffeinated beverages or foods 24 hours before the experimental procedure. Subjects were advised to wear comfortable clothes that are appropriate to exercise, and to eat a light meal two hours before the procedures.

Following recommendations from the American College of Sports Medicine (ACSM),^21^ to avoid dehydration of the participants during the exercise,^22^ participants were instructed to drink 500 mL of water two hours prior to the sessions.

In the first phase of the study, VO2max of each participant was determined. In the second phase, the subjects followed the placebo protocol (250mL water) or the ED protocol (250 mL ED) 15 minutes before the exercise. In the third phase, participants followed the alternative protocol to the previous stage. An independent researcher who did not participate in the study data collection provided the drinks. Both researchers and subjects were blinded to the sequence of interventions.

The ED (250mL) had an energy content of 45 kcal and was composed of 11.2 g of carbohydrates, 80 mg of sodium, 32 mg of caffeine, 400 mg of taurine, 4.6 mg of niacin, 2 mg of pantothenic acid, 0.5 mg of vitamin B6, 0.4 mg of vitamin B12, 240 mg of glucuronolactone, and 20 mg of inositol.^16^

The intensity of aerobic exercise in all stages was prescribed based on the VO2max of each participant. The treadmill test had a total duration of 30 minutes. First, the subjects walked on a treadmill at a speed of 5Km/h for five minutes of warm-up; the speed was increased to the corresponding 60% of VO2max for 25 minutes. Then, the subjects rested in the supine position for 60 minutes (recovery period).

#### Cardiorespiratory variables

The test to determine the VO2max was performed on a treadmill (TPEE; Inbrasport ATL 2000) using the Bruce protocol.^23^ The subjects remained at rest on the treadmill in an orthostatic position for stabilization of baseline cardiovascular values. Then, the stress test was initiated, with progressive increase in the workload by means of increased inclination and speed of the treadmill every three minutes. Verbal encouragement was given in an attempt to obtain maximum physical effort. The test was interrupted because of exhaustion or any clinical and/or electrocardiographic abnormality.

During the test, HR and subjective perception of effort were monitored at the end of each stage by the Borg Scale for perceived pain and effort.^24^ For the test to be recognized as maximum, subjects should attain 90% of maximum HR, earlier estimated (220 - age).^25^

The analysis of expired gases was conducted using the Quark PFT commercial system (Comend, Rome, Italy), and the VO2peak was defined as the highest VO2max attained during the test.

The subjects were split into two groups based on the median VO2 peak value:

1. Higher VO2 peak (HO) group, composed of subjects with peak VO2 > 52.15 mL/Kg/min, and
2. Lower VO2 peak (LO) group, composed of subjects with peak VO2 <52.15 mL/Kg/min.

#### Cardiovascular parameters

Cardiovascular parameters were measured with subjects in the supine position. Systolic blood pressure (SBP) and diastolic blood pressure (DBP) measures were taken by auscultation with a stethoscope (Littman Classic II^®^, St. Paul, USA) and a calibrated aneroid sphygmomanometer (Welch Allyn Tycos^®^, New York, USA) on the individuals’ left arm. HR was measured using a Polar RS800CX^®^HR monitor. Respiratory rate (RR) was determined by counting the subjects’ breaths for one minute, without the subjects being aware of it, so that no change in the breathing pattern occurred. Oxygen saturation (SpO2) was measured by pulse oximetry (PM-50 Mindray^®^, China).

#### HRV Analysis

HRV was measured according to the recommendations of the Task Force of the European Society of Cardiology and the North American Society of Pacing and Electrophysiology.^26^ The sensor chest strap was worn on the chest the Polar RS800CX heart rate receiver was placed on the left wrist. The HRV pattern was recorded beat by beat. The final 256 consecutive stable RR intervals of each recording were selected. Then, digital and manual filtrations were performed to eliminate artifacts and premature ectopic beats. Only series with an excess of 95% of sinus beats were included in the analysis.

The time-domain index of HRV was determined by the root mean square of successive differences (RMSSD) and standard deviation of the normalized N–N interval (SDNN). The frequency-domain index was evaluated by the high-frequency (HF) (0.15 to 0.4 Hz) and low-frequency components (LF) of the power spectral density (0.04 to 0.15 Hz) in milliseconds squared and absolute units, and ratio LF/HF (ms^2^). The Poincaré plot analysis was made using the SD1 (standard deviation of the instantaneous beat-to-beat variability) and SD2 (standard deviation of long-term continuous beat-to-beat variability).

The Kubios HRV^®^analysis software package was used to compute these indices.

#### Measurement of parameters

HR, RR, SBP, DBP and SpO2 were recorded at the following time points: rest – 15^th^minute after ED and placebo ingestion – and during recovery - 1^st^, 3^rd^, 5^th^, 7^th^, 10^th^, 20^th^, 30^th^, 40^th^, 50^th^and 60^th^minutes after exercise.

The HRV indexes were measured at the following time points: “rest” (15 to 20 minutes of resting after EB or placebo ingestion; and during “recovery”: Rec1 (zero to five minutes), Rec2 (five to ten minutes), Rec3 (15 to 20 minutes), Rec4 (25 to 30 minutes), Rec5 (35 to 40 minutes), Rec6 (45 to 50 minutes), and Rec7 (55 to 60 minutes).

#### Sample size

The sample size was calculated based on a previous study,^22^ which gave us the magnitude of the difference, and we calculated the RMSSD index as a reference. We determined a standard deviation of 16.2ms and the magnitude of the difference was 11ms. A minimum sample size of 14 subjects per group was calculated, with an alpha risk of 5% and beta risk of 80%.

## Statistical analysis

Data analysis and data reporting were conducted following the recommendations of Laborde *et al.* ^27^ Data normality was tested by the Shapiro-Wilk test. To compare cardiovascular variables and HRV, we performed the repeated-measures analysis of variance (ANOVA), followed by the Bonferroni post-test for parametric distributions or Friedman followed by Dunn’s post-test for non-parametric distributions. P-values <0.05 were considered significant. The analyses were performed using the IBM SPSS Statistics software version 22.0 (SPSS Inc., Chicago, IL, USA).

### Randomization and outcome assessment

With the aim of minimizing the selection bias, the subjects and the researchers were uninformed about the sequence of procedures. An investigator who did not participate in the study randomly assigned the participants the interventions. Researchers specialized in the field who did not participate in the collections were invited to assess the outcome. So, the outcome evaluators were blinded, allowing the study to be less susceptible to detection bias. Also, all outcomes were reported in full, decreasing the likelihood of reporting bias.

## Results

Thirty-five men were considered eligible for the study; 28 met the inclusion criteria and completed the study ( Figure 1 ).

**Figure 1:** The CONSORT flow diagram.

Table 1 describes the anthropometric characteristics and the responses obtained in the maximum effort test for the groups with the highest VO2peak (HO), and with the lowest VO2peak (LO).

**Table 1:** Anthropometric characteristics and VO2peak values of the study subjects

	High VO2 peak		Low VO2 peak	

	Mean ± SD	Min - Máx	Mean ± SD	Min - Máx
Age (y)	22.93 ± 2.62	[18 - 26]	25.29 ± 3.07	[21 - 29]
Height (m)	1.78 ± 0.08	[1.68 - 1.94]	1.81 ± 12.52	[1.65 – 1.93]
Mass (kg)	77.55 ± 6.92	[60 - 96]	89.48 ± 12.52	[63.30 – 107.50]
BMI (kg/m^2^)	24.46 ± 2.56	[20.05 - 29.41]	27.12 ± 3.07	[19.94 - 27.70]
VO2_peak_(ml/kg/min)	60.14 ± 6.43	[52.40 - 77.77]	41.76 ± 10.14	[23.03 – 29.94]

*Y: years; m: meters; kg: kilogram; BMI: body mass index.*

In relation to HRV frequency-domain and HRV indexes, we detected a time effect (p=0.0001). No protocol interaction effect was seen for LF (normalized units, n.u.) (p=0.880), HF (n.u.) (p=0.163) and LF/HF ms^2^(p=0.086) indexes. No protocol effect was observed for LF (n.u.) (p=1.000), HF (n.u.) (p=0.675) and LF/HF (p=0.531). For LF (n.u,) index significant differences were achieved between rest and Rec1 in HO and LO in the ED protocols. There were significant differences in HF (n.u.) between rest and Rec1 for HO in the placebo protocol, and HO and LO during the ED protocol. Regarding the LF/HF ratio, significant differences were found between rest and Rec1 in HO and LO during the ED protocol. The response of frequency-domain HRV indexes are shown in Figure 2 .

**Figure 2:** Response of the frequency-domain heart rate variability indices at rest and during recovery from exercise in the groups of subjects with high VO2 peak (HO) and low VO2 peak (LO) receiving energy drink (energy) or placebo (Control).

SDNN and SD2 indices showed significant differences in the time effects (SDNN: p=0.0001; SD2: p=0.0001) and protocol interaction (SDNN: p<0.0001; SD2: p=0.0002), and only SDNN showed differences between protocols (SDNN: p=0.015; SD2 p=0.061). Significant differences in SDNN index were seen between rest and Rec1 in the LO group during the control protocol, and in RMSSD between Rest and Rec1 in both placebo and ED protocols.

Regarding RMSSD and SD1 indexes we detected significant differences in time effects (RMSSD: p<0.0001; SD1: p<0.0001), protocol interaction (RMSSD: p=0.009; SD1: p=0.036) and between protocols (RMSSD: p=0.025; SD1=0.010). Significant changes for the time domain were observed between rest and Rec1 for RMSSD index and SD1 index for all protocols. Significant differences for the time domain were found between rest and Rec2 in the HO group in the control protocol and in the LO group in the ED protocol for SD1 index. Figure 3 displays the response of time-domain HRV indexes at rest and during recovery from exercise.

**Figure 3:** Time-domain heart rate variability indexes at rest and during recovery from exercise in the groups of subjects with high VO2 peak (HO) and low VO2 peak (LO) receiving energy drink (energy) or placebo (Control)

In relation to cardiorespiratory parameters, we observed a time effect (p=0.0001) for HR, RF, SBP, DBP (p=0.0001) and no effect was observed in SpO2 (p=0.188). No significant protocol interaction effect was seen for SBP, DBP, RF or SpO2 (SBP: p=0.424; DBP: p=0.259; RF: p=0.340; SpO2: p=0.346), but a significant effect was seen for HR (p<0.0001). Significant differences were achieved between protocols for SBP, DBP and HR (SBP: p=0.001; DBP: p=0.014; HR: p=0.011) and no difference was found for RF and SpO2 (RF: p=0.132; SpO2: p=0.083). Significant differences in HR and SBP were seen in the time domain between rest and Rec1 for all protocols. Figure 4 displays the response of cardiorespiratory parameters at rest and during recovery from exercise.

**Figure 4:** Cardiorespiratory parameters at rest and during recovery from exercise in the groups of subjects with high VO2 peak (HO) and low VO2 peak (LO) receiving energy drink (energy) or placebo (Control). HR: high frequency; RF: Respiratory frequency.; SBP: systolic blood pressure; DBP: diastolic blood pressure.

## Discussion

Our study was undertaken to evaluate the impact of ED on HRV and cardiovascular recovery after exercise in individuals with different cardiorespiratory fitness. As key findings, we reported that ED before exercise did not influence SBP, DBP, SpO2 or RF in the post-exercise recovery, and delayed the LF and LF/HF recovery following effort.

Constituents such as caffeine, taurine, glucuronolactone, B vitamins, guarana, ginseng, ginkgo biloba, l-carnitine, sugars, antioxidants, and trace elements are usually found in EDs.^28^ Caffeine stimulates the central nervous system via the activation of the sympathetic adrenal-medullary system, raising blood pressure in situations of psychological^29^ and physiological stress, for instance physical exercise.^30 , 31^

Cardiovascular adjustments are required to maintain adequate perfusion to other organs.^32^ When the exercise begins, the central command resets the levels of the arterial baroreflex, resulting in lessened parasympathetic conduction, and light reduction in the ANS activity because of the venous return in this first phase.^33^

The upsurge in reflexive amplitude via the early increase in HR is caused by the increased load on pulmonary baroreceptors, which allows the parasympathetic nervous system to cut its cardiac activity. As the workload increases, the central command increases and readapts the arterial baroreflex. Therefore, there is a depression of the parasympathetic reflex response, increase in the sympathetic nervous system, increasing HR and cardiac contraction strength.^34^

There are reports in the scientific literature that indicate an intimate connection between ED and changes in the cardiovascular system. ED depresses the parasympathetic nervous system and/or increases the sympathetic nervous system in obese young people,^35^ increases SBP,^36^ changes nonlinear HRV in young adults,^37^ and delays HR and HRV post-exercise recovery when mixed with alcohol.

Recently, our group reported that ED is unable to postpone the HR recovery after exercise.^15^ In the cited study, 29 healthy men between 18 and 30 years old performed aerobic exercise after consuming ED or placebo. There was an important reduction in HRV in the initial five minutes after exercise in both protocols. So, the main conclusion was that ED was unable to influence post-exercise HR recovery.^15^ In another study with similar protocols, An *et al.* ^16^ found no significant fluctuations in these parameters between the interventions, suggesting no significant effect of ED.

In another randomized, crossover, placebo-controlled clinical trial, 15 (eight men) young adults who were physically active were evaluated for the effects of ED.^17^ After fasting for eight hours, they consumed standardized ED (2mg/kg of caffeine) or placebo with a similar taste.^17^ After submaximal aerobic exercise for 30 minutes, these individuals were induced to fatigue by pedaling 10 minutes at 80% of the ventilatory threshold. Resting HR was higher when subjects consumed ED, when compared to placebo (ED: 65+10bpm vs. Placebo: 58+8bpm, p=0.02), but HRV indices (RMSSD, SDNN, PNN50, HF, LF and LF/HF) were unchanged.^17^

In the double-blind, crossover, counterbalanced and placebo-controlled study by Clark et al.^14^ 17 (10 women) young adults were exposed to a graded test of exhaustion on an exercise bike after ingestion of 140mg of caffeine or placebo. HRV data were recorded before, during and after 15 minutes of physical exercise. Substantial increases in HF and RMSSD indices were detected in the ED group during exercise. A sub analysis between genders demonstrated changes in the initial RMSSD values and in the amount of decline. The consumption of ED was able to sex-dependently affect cardiac autonomic responses during low-, moderate- and high-intensity exercise. However, in the post-exercise, no differences were found in HR recovery after ED ingestion.

It is crucial to emphasize that these research studies did not take into account the cardiorespiratory capacity of the subjects. A more recent study^13^ evaluated the impact of caffeine on post-exercise HR recovery in men with different VO2. The authors split young male adults into two groups based on their VO2: (1) high VO2 (HO): 16 volunteers, peak VO2> 42.46 mL/kg/min; and (2) low VO2 (LO): 16 individuals, VO2 <42.46 mL/kg/min). The subjects participated in two intervention protocols, with ingestion of capsules containing 300mg of starch (placebo protocol) or 300 mg of caffeine (caffeine protocol). After the ingestion of caffeine or placebo, participants rested for 15 minutes, and then were submitted to 30 minutes of exercise on a treadmill at 60% of VO2 peak. HRV indices in the time and frequency domains disclosed significant changes for the RMSSD and SDNN indices in the recovery between groups (p<0.001). Remarkable adjustments were observed (rest *versus* recovery) from the 0 to the 5^th^minute of recovery from exercise for the LO group in the placebo protocol and from the 5^th^minute to the 10^th^minute of recovery for the LO in the caffeine protocol. In our study, significant deviations were detected only in the first five minutes of recovery in the HO individuals in both protocols. These data corroborate that caffeine delays parasympathetic recovery from exercise in individuals with lower cardiorespiratory capacity.^13^

Concerning the cardiorespiratory parameters, no significant changes were found that would suggest different effects of ED in individuals with different cardiorespiratory capacities. These findings corroborate the study conducted by An et al.,^16^ where no significant changes were revealed in HR and blood pressure during recovery after maximum exercise, after ingestion of ED in different concentrations (1.25 to 2.5 mg/Kg).

It has been suggested that the effect of ED on the cardiovascular system may be dose-related. In the study by Shah et al.,^35^ consumption of ED in high doses (32 ounces, equivalent to 946.3 mL) resulted in a significant and prolonged increase in the QTc interval, SBP and DBP as compared with placebo in healthy young people.

Regarding parameters that reflect the respiratory component, for instance SpO2 and RF, no significant differences were found in our study. In both protocols, all individuals showed adequate values of these variables, as would be expected for healthy subjects without recognized cardiopulmonary diseases.^11^

Finally, considering that we detected slightly delayed HR recovery in both groups that ingested ED, our data draws attention to subjects with cardiovascular and metabolic diseases who consume EDs (as a supplement) before exercise.

### Strengths and limitations of the study

One of the strengths of this study concerns is methodology. Although we did not assess plasma catecholamine concentrations or sympathetic nerve activity, we evaluated HRV, a simple, reliable, non-invasive method and a significant quantitative marker for estimating autonomic HR modulation.^9^ The sample was comprised of healthy young people, with the aim of avoiding the influence of sex hormones. For this reason, our results cannot be applied to females or subjects taking medications that could that affect the ANS. Nonetheless, the study design and performance of rigorous procedures to avoid selection, detection, attrition and reporting biases support our results. Our study provides relevant information about the mechanisms linked to the impact of ED on post-exercise recovery.

## Conclusions

Acute ED intake delayed HR recovery following exercise in subjects with low and high cardiorespiratory fitness.
